# Blood-based biomarkers and early diagnosis of Alzheimer’s disease

**DOI:** 10.4103/NRR.NRR-D-25-00759

**Published:** 2026-01-27

**Authors:** Zhikang Cui, Guixia Li, Shuyong Wei, Qian Cheng, Qian Yu, Shuai Zong, Pengfei Zhang, Hang Chen, Shuyi Yu, Shuang Wu, Ming Li, Zhiming Lu

**Affiliations:** 1 Department of Laboratory Medicine, Shandong Provincial Hospital Affiliated to Shandong First Medical University, Jinan, Shandong Province, China; 2 Department of Laboratory Medicine, Heze Municipal Hospital, Heze, Shandong Province, China; 3 Xiajin County People’s Hospital, Dezhou, Shandong Province, China; 4 Department of Laboratory Medicine, Shandong Provincial Hospital, Cheeloo College of Medicine, Shandong University, Jinan, Shandong Province, China

**Keywords:** Alzheimer’s disease, amyloid plaques, biomarkers, dementia, early diagnosis, nerve regeneration, neurodegenerative diseases, neurofibrillary tangles, neuroinflammation, neuropathology

## Abstract

Alzheimer’s disease is a common neurodegenerative disease characterized by progressive memory loss, cognitive decline, and behavioral changes. Blood-based biomarkers have recently gained significant attention due to their accessibility and cost-effectiveness. This review highlights the latest progress in multiple key areas of blood-based biomarkers for Alzheimer’s disease. For early diagnosis, blood-based biomarkers such as amyloid-β and phosphorylated tau can identify Alzheimer’s disease even before clinical symptoms emerge. Dynamic changes in blood-based biomarkers, including p-tau217 and neurofilament light chain, reflect disease progression and correlate with cognitive decline, enabling continuous monitoring of Alzheimer’s disease progression. Additionally, blood-based biomarkers such as p-tau181 and glial fibrillary acidic protein aid in differential diagnosis by distinguishing Alzheimer’s disease from other dementias such as frontotemporal dementia. Blood-based biomarkers related to nerve repair have opened up new avenues for tracking nerve regeneration and therapeutic response, especially brain-derived neurotrophic factor. Furthermore, advanced detection technologies such as single-molecule array and immunoprecipitation-mass spectrometry have significantly improved the sensitivity and specificity of blood-based biomarkers, facilitating their clinical translation. In summary, blood-based biomarkers hold strong potential to improve early diagnosis, monitor progression, differential diagnosis, and evaluate therapies in Alzheimer’s disease. This review provides a comprehensive and updated evaluation of the translational potential of blood-based biomarkers, emphasizing their practical utility in clinical settings and offering insights into future directions for large-scale application. This review emphasizes the need to prioritize the allocation of scientific resources, expedite the transition of blood-based biomarkers to clinical implementation, and ultimately achieve precise treatment of Alzheimer’s disease using these biomarkers.

**Facts**

• A biological definition of Alzheimer’s disease has been successfully established, centered on amyloid-β and tau proteins as core pathological biomarkers, marking a paradigm shift from clinical symptom diagnosis to biomarker definition.

• The high correlation between blood biomarkers for Alzheimer’s disease and results from cerebrospinal fluid and positron emission tomography imaging has been validated, laying a scientific foundation for the development of non-invasive and accessible early detection tools.

• Based on the precise screening strategy for Alzheimer’s disease biomarkers, successful applications have been made in key clinical trials of disease-modifying therapies such as lecanemab and donanemab, demonstrating the clinical value of early biomarker intervention.

**Open questions**

• What key standardization and validation challenges need to be addressed to achieve the large-scale application of brain biomarkers in primary healthcare and community medicine?

• How can we optimize and integrate different blood–brain barrier biomarkers to construct a comprehensive model that accurately predicts individual disease progression and treatment response?

• As disease-modifying therapies targeting the pathophysiology of Alzheimer’s disease emerge, what specific roles will blood–brain barrier biomarkers play in monitoring treatment efficacy, identifying side effects, and guiding personalized treatment plans?

## Introduction

The World Alzheimer Report 2024 indicates that over 55 million individuals globally currently live with dementia, with Alzheimer’s disease (AD) accounting for approximately 60%–70% of cases. The clinical manifestations of AD include cognitive impairment caused by episodic memory impairment, decreased learning ability, and language deficits (Dolphin et al., 2024). The clinical progression of AD progresses from the preclinical asymptomatic stage to mild cognitive impairment (MCI) due to AD, ultimately leading to dementia with irreversible neuronal loss (Tahami Monfared et al., 2022). In recent years, multiple AD treatment drugs have entered clinical trials, and disease-modifying therapies (DMTs) have reignited hope for effective intervention in AD (Tahami Monfared et al., 2023). Early diagnosis and intervention are critical to various treatment strategies for AD. Accurately identifying high-risk individuals enables DMTs to play a role in the early stages of AD disease. Although there is no clinical requirement to detect AD in cognitively normal (CN) individuals, this may change with the advancement of anti-amyloid-β(Aβ) therapy trials. Biomarkers are essential for evaluating drug efficacy, personalizing treatment, and slowing disease progression. Biomarkers could also be considered in subjective cognitive decline (SCD) patients with gradual, yet otherwise normal, deterioration of mental function (Hansson et al., 2023).

**Figure 1:**
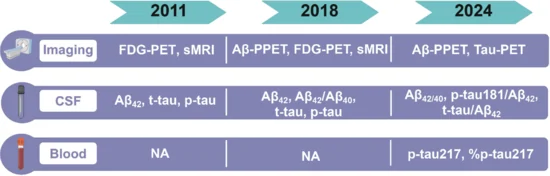
Diagnostic criteria for AD according to NIA-AA. The NIA-AA published diagnostic criteria for AD that include imaging, cerebrospinal fluid (CSF), and blood-based biomarkers (BBMs) used for diagnosis. These criteria were updated in 2011, 2018, and 2024, respectively. The imaging and CSF biomarkers utilized for diagnosis have evolved over time. Notably, BBMs were included in the updated 2024 criteria for diagnosing AD. Created in BioRender. Cui, Z. (2025) https://BioRender.com/put1olm. AD: Alzheimer’s disease; Aβ: amyloid-β; Aβ-PET: Aβ positron emission tomography; BBMs: blood-based biomarkers; CSF: cerebrospinal fluid; FDG-PET: fluorodeoxyglucose positron emission tomography; GFAP: glial fibrillary acidic protein; NA: not available; NfL: neurofilament light chain; NIA-AA: National Institute on Aging Alzheimer’s Association; p-tau: phosphorylated tau; sMRI: structural magnetic resonance imaging; tau-PET: tau-protein positron emission tomography.

The diagnostic and staging criteria for AD published by the National Institute on Aging-Alzheimer’s Association (NIA-AA) reemphasize the use of biomarkers to define AD rather than relying solely on clinical symptoms (Jack et al., 2024). **Figure 1** illustrates the development and changes in biomarkers released by the NIA-AA for diagnosing AD. Increasing attention is being given to diagnosing AD through biomarkers instead of relying solely on clinical symptoms. Core biomarkers include Aβ-related biomarkers (A), such as amyloid-β positron emission tomography (Aβ-PET), and tauopathy-related biomarkers (T), which are subdivided into early-stage markers (T1) such as p-tau217 and p-tau181, and later-stage markers (T2) such as MTBR-tau243. Non-specific markers include neurodegeneration biomarkers (N), such as neurofilament light chain (NfL), and inflammatory biomarkers (I), such as glial fibrillary acidic protein (GFAP). Additionally, biomarkers of non-AD comorbidities include vascular injury biomarkers (V) and α-synucleinopathy biomarkers (S) (Jack et al., 2024). Furthermore, diagnosing and treating nerve regeneration and repair are emerging fields in the diagnosis and treatment of AD, and blood-based biomarkers (BBMs) have opened new horizons for reflecting nerve regeneration and repair, such as brain-derived neurotrophic factor (BDNF).

To date, the diagnosis of AD faces numerous challenges, primarily due to the lack of sensitive and specific biomarkers. While cerebrospinal fluid (CSF) analysis and positron emission tomography (PET) imaging are considered gold standards for detecting AD-related pathology, these methods are costly and technically complex, limiting their feasibility for large-scale screening and early diagnosis. In recent years, scientific attention has increasingly turned to BBMs to enable more straightforward and economical approaches for AD diagnosis and monitoring (Abbott, 2024). BBMs are more favorable than invasive and costly diagnostic methods such as neuroimaging and spinal fluid analysis. They can identify Aβdeposition and tau pathology in the brain in a non-invasive manner at early stages, even before the appearance of clinical symptoms. Two anti-Aβ antibody drugs that have received regulatory approval, namely lecanemab (Van Dyck et al., 2023) and donanemab (Sims et al., 2023), utilized BBMs to screen participants in their clinical trials. BBMs, in particular, have shown promise as accessible, cost-effective methods for early AD detection, making them practical tools for broader screening and early intervention. Although standardization and specificity still need to be addressed, BBMs (such as p-tau217 combined with Aβ_42/40_) are close to clinical transformation. In the future, they may promote precise diagnosis, risk stratification, and treatment evaluation for AD, providing a key basis for early intervention. Moreover, with the development of AD BBMs, there is potential for more comprehensive monitoring of the diverse pathological processes associated with AD, paving the way for the optimization of personalized treatment strategies

This review aims to integrate the latest advances in the pathogenesis of AD, BBMs, and DMTs, focusing on the clinical translational potential of BBMs. The comprehensive integration of multimodal BBMs will contribute to the clinical translation of AD, particularly concerning early disease diagnosis. Unlike previous studies that focused on individual biomarker categories or the unilateral effects of biomarkers, this article offers a new evaluative perspective on how BBMs can guide resource allocation and clinical decision-making. Additionally, we emphasize emerging evidence and methodological innovations that support the use of BBMs as practical tools for precision medicine in AD, providing actionable insights into the application of BBMs for researchers and clinicians.

## Search Strategy

For this review on BBMs in AD, we searched PubMed/MEDLINE using the Advanced Search Builder with keyword combinations including “(Alzheimer’s disease) AND (blood-based biomarker),” “(Alzheimer’s disease) AND (plasma biomarker),” and specific biomarker terms such as Aβ, tau, neurofilament light chain, GFAP, and BDNF, alongside methodological terms such as single molecule array (SIMOA) and mass spectrometry. For therapeutics, we used the search string “(Alzheimer’s disease) AND (disease-modifying therapy OR monoclonal antibody OR lecanemab OR donanemab).” A systematic search was conducted that combined keywords, Medical Subject Headings, and Boolean operators across databases. Additionally, backward and forward citation tracking of seminal papers ensured comprehensive coverage. Retrieved articles were manually screened based on title and abstract, prioritizing studies that validated BBMs in AD cohorts, examined longitudinal changes, compared diagnostic accuracy with established biomarkers, or addressed clinical translation challenges, while excluding non-blood biomarkers, non-human studies, and redundant publications. All years were included in the search. Finally, this review contains 330 references to support our viewpoint.

## Blood-based Biomarkers for Early Diagnosis of Alzheimer’s Disease

**Figure 2:**
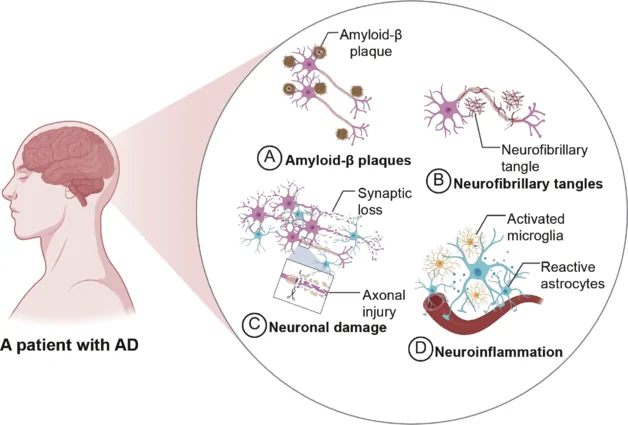
Neurological pathological mechanism diagram of AD. The neuropathological changes in AD include (A) amyloid-β plaques, (B) neurofibrillary tangles, (C) neuronal damage, and (D) neuroinflammation. Created in BioRender. Cui, Z. (2025) https://BioRender.com/34zems1. AD: Alzheimer’s disease.

The clinical course of AD usually lasts for 8–10 years. However, there is a long period before the onset of symptoms, which may last more than 20 years, characterized by a gradual accumulation of pathological changes. AD is a neurodegenerative disease. **Figure 2** shows its main neurological pathological mechanisms, including the deposition of Aβ plaques, neurofibrillary tangles (NFTs) caused by excessive phosphorylation of tau (p-tau) protein, neuroinflammation, and loss of sex neurons and synapses(Ju and Tam, 2022). Timely detection of AD is essential for effective management and treatment (Masters et al., 2015), particularly given the recent advancements in Aβ-targeted therapies like lecanemab and other investigational drugs designed for early-stage AD or MCI patients (Sims et al., 2023; Van Dyck et al., 2023). The Food and Drug Administration (FDA)has authorized two *in vitro* diagnostic tests for identifying Aβ plaques linked to AD. These tests include Lumipulse, which evaluates the Aβ_42/40_ ratios in CSF, and Elecsys, which measures p-tau181/Aβ_42_ and t-tau/Aβ_42_ ratios, both aiding in determining whether cognitive decline is due to AD. Research has shown that AD-related pathological changes begin more than 20 years before typical symptoms appear. These changes first manifest as the deposition of Aβ plaques in certain specific brain regions, such as the posterior cingulate cortex, prefrontal cortex, and temporoparietal area. The accumulation of Aβ plaques in these areas can lead to metabolic disorders, tau pathology, and hippocampal atrophy, ultimately resulting in clinical symptoms such as cognitive decline (Jia et al., 2024).

### Amyloid-β related blood-based biomarkers

#### Amyloid-β 42/40

The abnormal deposition of Aβ plaques and NFTs is a key biomarker for diagnosing AD, and changes in Aβ metabolism are considered one of the earliest pathological changes in AD (Guo et al., 2021). The accumulation of Aβ can lead to permanent damage to neurons. Intervention in Aβ metabolism before significant neuronal damage may be optimal for treating AD (Kang et al., 1987). The APP mainly splits into Aβ_42_ and Aβ_40_, which are approximately 4 kDa in size and are the main components of AD plaques (Kang et al., 1987). The accumulation of Aβ plaques is considered an early event in the pathogenesis of AD, followed by excessive phosphorylation of tau protein (Trelle et al., 2024). The presence of Aβ can lead to excessive local brain activity and significantly increase electroencephalogram (EEG) activity (Kang et al., 1987). As the disease progresses and tau protein accumulates, the neurophysiological activity of the brain gradually slows down, and the combined deposition of Aβ and tau further exacerbates the slowing down of brain activity (Gallego-Rudolf et al., 2024).

In recent years, as the need for early detection and intervention in AD has grown, significant progress has been made in clinical research on plasma Aβ_42/40_; alterations in this ratio are believed to indicate the development of Aβ plaques within the brain (Masters et al., 2015). Extensive research has demonstrated a strong correlation between plasma Aβ_42/40_ levels and the neurodegenerative alterations observed in AD (Bermudez et al., 2023), particularly in accurately predicting neuroinflammatory plaque scores (Bermudez et al., 2023). Low baseline plasma Aβ_42/40_ is an independent predictor of future increases in Aβ-PET signals (Pereira et al., 2021), and its performance is comparable to CSF Aβ_42/40_. In a study by Bun et al. (2023) that utilized a high-sensitivity chemiluminescence immunoassay, the area under the curve (AUC) of plasma Aβ_42/40_ levels to distinguish Aβ-PET positive individuals was 0.949. These findings support the potential of plasma Aβ_42/40_ as an early diagnostic biomarker for AD. However, it is important to note that although plasma Aβ_42/40_ levels in AD patients decrease, the magnitude of these changes is relatively small, which limits their diagnostic efficacy as an independent biomarker. The combination of plasma Aβ_42/40_ and p-tau217 can improve the accuracy of BBMs in predicting Aβ-PET and can even predict the early accumulation of Aβ in AD patients below the Aβ-PET threshold. Therefore, it is necessary to include plasma Aβ_42/40_ in clinical trials and early screening for AD (Janelidze et al., 2024).

#### Amyloid-β oligomers

Amyloid-β oligomers (AβOs) are products formed by the aggregation of Aβ monomers and are a key factor in the pathogenesis of AD. AβOs can diffuse throughout the brain and inhibit the synaptic function of neurons (Tolar et al., 2021). They interact with receptors on the surface of neurons, altering the activities of intracellular signaling pathways (Viola and Klein, 2015), which affects neurotransmission and leads to cognitive impairments (Li et al., 2009). Furthermore, Aβ oligomers promote the abnormal phosphorylation of tau proteins, forming NFTs, another pathological hallmark of AD (Nisbet et al., 2015).

AβOs can be divided into low and high molecular weight forms. Low molecular weight forms include dimers and trimers, while high molecular weight forms include Aβ*56 and fibrils. Among these, Aβ dimers are the focus of researchers’ attention, as their levels in the brains of AD patients are significantly elevated. In the early stages of AD, the plasma AβO levels in patients substantially increase, which is closely related to the accumulation of Aβ in the brain. The increase in AβOs is associated with memory decline, particularly with the deterioration of episodic memory (Meng et al., 2019). In cognitively healthy individuals, many with subjective cognitive decline (SCD) show higher plasma AβO levels, even without a corresponding increase in brain Aβ accumulation (Kim et al., 2022). Therefore, plasma AβOs could serve as biomarkers for the early diagnosis of AD.

The multimer detection system-oligomeric amyloid-β (MDS-OAβ) detection system is an enhanced enzyme-linked immunosorbent assay (ELISA) technique designed to detect Aβ oligomers rather than monomers in plasma (BabapourMofrad et al., 2021). This technology can measure the dynamic changes in the concentration of Aβ oligomers and is currently used to detect AD-related biomarkers. The trend of Aβ protein oligomerization in plasma is positively correlated with the severity of AD and Aβ deposition in the brain (Wang et al., 2024a). MDS-OAβ has high predictive accuracy (AUC = 0.855) in distinguishing early Aβ-positive PET scans, and its accuracy is further improved when combined with other clinical data (Pyun et al., 2021).

### β*-Site amyloid precursor protein cleaving enzyme 1*

β-Site amyloid precursor protein cleaving enzyme 1 (BACE1) hydrolyzes the APP protein to produce Aβ peptide, which constitutes the main component of Aβ plaques in the brains of AD patients. BACE1 catalyzes the initial and rate-limiting step in APP processing (Vassar, 2004). BACE1 is predominantly found in cellular membranes and endosomal structures, and it is also present in functional synaptic terminals and aberrant neuronal projections surrounding Aβ plaques (Hampel et al., 2021a). Excessive activation of BACE1 is strongly linked to the key pathophysiological characteristics of AD (Tijms et al., 2018).

Many studies have focused on serum BACE1, revealing that patients with MCI who demonstrate better cognitive performance show higher serum BACE1 activity, which correlates with the progression to dementia (Cervellati et al., 2022; Ihara, 2022). Analytical methods for measuring BACE1 activity include electrochemical biosensors, fluorescence biosensors, and inductively coupled plasma mass spectrometry (Fan et al., 2024). A study using a luciferase assay to measure serum BACE1 activity revealed that BACE1 levels were more than 60% higher in AD patients than in healthy controls (Nicsanu et al., 2022). Furthermore, BACE1 showed superior diagnostic accuracy over the Aβ_40/42_ ratio, suggesting that dysregulation of BACE1 may be a potential blood biomarker for early AD diagnosis (Saraceno et al., 2024). All these findings suggest that detecting BACE1 activity in serum could be a potential biomarker for the early diagnosis of AD.

### Phosphorylated tau related blood-based biomarkers

During the progression of AD, tau hyperphosphorylation triggers conformational changes that convert soluble tau into NFTs (Hansson, 2021). Current *in vivo* biomarkers for the stages of NFT development include soluble phosphorylated tau (p-tau) levels at multiple phosphorylation sites in CSF and plasma (Leuzy et al., 2021), alongside tau-PET tracers that quantify insoluble cerebral tau aggregates (Wolters et al., 2021). Pathological tau phosphorylation in AD impairs microtubule binding, leading to intracellular accumulation and NFT formation. Pathological tau protein can be phosphorylated at multiple amino acid sites, including 181, 199, 202, 205, 217, 231, 235, and 396 (Lantero-Rodriguez et al., 2024). Plasma p-tau181, p-tau217, and p-tau231 are pivotal early-detection biomarkers that capture cerebral tau and Aβ pathology years before clinical manifestation.

#### Phosphorylated tau181

Elevated levels of phosphorylated tau181 (p-tau181) in CSF and plasma strongly correlate with tau pathology associated with AD (Basheer et al., 2023). Plasma p-tau181 shows a progressive elevation across the AD continuum (Wojdała et al., 2023), with significant increases observed in the early stages of AD and minimal changes in non-AD dementias. In a cohort study involving patients with SCD and MCI (Giacomucci et al., 2023), elevated plasma p-tau181 levels were found to be closely associated with AD pathology but not with cognitive status. A comparative study of plasma p-tau181 with PET imaging revealed that plasma p-tau181 had an AUC of 0.93 for distinguishing Aβ-positive (Aβ^+^) from Aβ-negative (Aβ^–^) individuals and was comparable to PET imaging in predicting Aβ status (Lu et al., 2023). Simrén et al. (2021) further confirmed the superiority of plasma p-tau181 for the early diagnosis of AD, reporting an AUC of 0.91 for distinguishing AD from cognitively normal individuals, which is significantly higher than that of other biomarkers. However, some researchers have found that while plasma p-tau181 accurately reflects the CSF A^+^/T^+^ status in AD dementia and AD-induced MCI, it fails to do so in asymptomatic AD, which may limit its use as an early screening biomarker (Bellomo et al., 2024). It is also noteworthy that p-tau181 levels in plasma often increase with age, suggesting that age should be considered when determining the biological reference range for plasma p-tau181 (Bellomo et al., 2024).

#### Phosphorylated tau217

Since its discovery, phosphorylated tau217 (p-tau217) has emerged as a prominent candidate biomarker for AD (Telser et al., 2022). Plasma p-tau217 levels increase as early as the preclinical or MCI stages of AD. In a study comparing various types of plasma tau proteins using immunoprecipitation-mass spectrometry (IP-MS), the plasma p-tau217 level in AD patients was found to be approximately 2.77 times higher than that in non-AD patients, representing a greater increase than that observed with other biomarkers (Montoliu-Gaya et al., 2023). A recent study has shown that plasma p-tau217 has a positive predictive value (PPV) greater than 95% for distinguishing preclinical AD dementia and effectively detecting Aβ pathology (Therriault et al., 2024).

**Figure 3:**
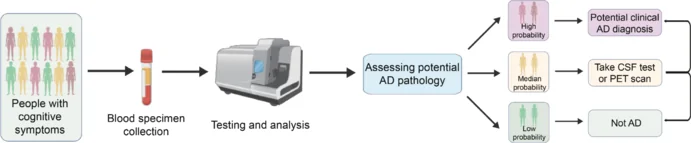
Potential BBM-based workflow for AD diagnosis. Based on the current clinical trial status, the two-step diagnosis workflow for AD using BBMs is highly recommended. The first step involves utilizing highly sensitive BBMs, such as p-tau217 and the Aβ_42_/Aβ_40_ ratio, for the initial screening of suspected patients. This approach enables the rapid identification of low-risk individuals, thereby avoiding unnecessary CSF analysis or PET scans and improving the allocation efficiency of diagnostic resources. Patients classified as medium or high risk undergo a stratified evaluation in the second step. Medium-risk patients require further confirmatory testing, such as CSF analysis or PET scans, to clarify pathological features, while high-risk patients can proceed directly to the diagnosis stage. Created with BioGDP (BioGDP.com). AD: Alzheimer’s disease; BBMs: blood-based biomarkers; CSF: cerebrospinal fluid; PET: positron emission tomography; p-tau217: phosphorylated tau217.

Previous research has indicated that plasma p-tau217 levels can be reliably detected with only 4 mL of blood, with diagnostic accuracy ranging from 89% to 98% (Palmqvist et al., 2020). A study involving 81 patients with early-onset or atypical dementia found that plasma p-tau217 and [^18^F] FDG-PET had similar specificity in diagnosing AD (70%). In contrast, plasma p-tau217 demonstrated higher sensitivity (97% *vs.* 73%) and overall accuracy (AUC 84% *vs.* 72%) (Quispialaya et al., 2025). A blood model based on plasma p-tau217 levels can calculate the probability of Aβ-PET positivity and stratify patients into low-, medium-, and high-risk categories. Subsequently, patients in the medium-risk group undergo Aβ_42/40_ ratio testing in CSF. Utilizing the plasma p-tau217 model, this two-step screening approach provides a cost-effective strategy for AD detection, particularly in memory clinics, and highlights the significant potential of p-tau217 for early AD diagnosis (Brum et al., 2023). **Figure 3** shows the workflow for diagnosing AD based on biomarkers, which is expected to be applied clinically.

Hybrid ratios diminish inter-sample variability, improve measurement precision, and more accurately reflect pathological changes by minimizing nonspecific fluctuations. The plasma p-tau217 to non-phosphorylated tau hybrid ratio (%p-tau217) demonstrates significant potential for AD diagnosis. A large-scale analysis of 1422 plasma samples from the Swedish BioFINDER-2 cohort revealed outstanding performance by plasma %p-tau217 in classifying Aβ-PET status (AUC = 0.97), achieving statistical equivalence to CSF p-tau181/Aβ42 (AUC = 0.97) and CSF Aβ_42/40_ (AUC = 0.96) (Barthélemy et al., 2024). Furthermore, plasma %p-tau217 effectively classified Aβ-PET positivity in cognitively unimpaired individuals (AUC = 0.96). Subsequent studies have confirmed these findings, establishing the superiority of p-tau217 for determining Aβ status and enabling early AD diagnosis (Telser et al., 2022; Janelidze et al., 2024).

Plasma p-tau217 exhibits exceptional potential for early AD diagnosis, including the identification of patients suitable for anti-AD therapies (Yu et al., 2023). Future research should continue to explore changes in plasma p-tau217 during AD pathogenesis and its diagnostic role to better understand disease progression, maximize testing value, and refine detection technologies for early diagnosis (Therriault et al., 2025).

#### Phosphorylated tau231

Plasma p-tau231 levels increase during early AD stages, particularly in the neuropathological change phase (Smirnov et al., 2022) and brain regions associated with Aβ accumulation (Smirnov et al., 2022). Previous research utilizing different plasma tau proteins to predict AD pathological changes indicated that plasma p-tau231 may function as a biomarker across multiple disease stages, especially in early phases related to Aβ plaques (Montoliu-Gaya et al. 2023). A cohort study observed significant alterations in all tested biomarkers during AD preclinical stages (Milà-Alomà et al., 2022). Among these, p-tau231 distinguished itself by reaching abnormal levels at minimal Aβ load, demonstrating its capacity to detect early brain Aβ changes before evident Aβ plaque pathology.

p-tau231 rises earlier than p-tau181 and can forecast AD pathology before Aβ-PET scans become positive (Gonzalez-Ortiz et al., 2023). The plasma form of p-tau231 shows the strongest correlation with the Aβ-PET signal, particularly at early Aβ deposition sites such as the anterior cingulate, suggesting a close relationship with Aβ pathology (Meyer et al., 2022). This evidence indicates that plasma p-tau231 holds significant diagnostic value in prodromal AD, especially before the onset of clinical symptoms. Further research utilizing SIMOA technology has validated p-tau231 as a key parameter for early AD diagnosis, differential diagnosis, and as a reliable indicator of cerebral tau pathology (Pilotto et al., 2024).

### Neuronal damage related blood-based biomarkers

Neuronal damage occurs through multiple mechanisms. AβOs toxicity, microtubule damage from tau protein hyperphosphorylation, and mitochondrial dysfunction-induced apoptotic pathways all contribute to neuronal damage in patients with AD. Neuronal injury also leads to various forms of cell death, including apoptosis, pyroptosis, and ferroptosis. NfL and total tau (t-tau) are widely used biomarkers for neuronal injury in clinical and research settings. While these biomarkers indicate neuronal damage, dysfunction, or degeneration, they lack specificity for AD and may also be present in other neurological disorders.

#### Neurofilament light chain

NfL is a key cytoskeletal protein within neuronal axons, essential for maintaining neuronal structure, supporting intracellular transport, and facilitating neural signal transmission. Following axonal damage, NfL is released into CSF and blood (Hampel et al., 2023), resulting in significant concentration elevations. Research demonstrates that elevated NfL levels strongly correlate with neuronal injury across multiple neurodegenerative disorders (Assfaw et al., 2024) and exhibit an inverse relationship with overall cortical thickness (Lee et al., 2022), establishing NfL as a promising biomarker for neurodegeneration. Although increased NfL levels lack diagnostic specificity for AD, they may also be elevated in other neurodegenerative conditions (Koivumäki et al., 2024), including Lewy body dementia (Pilotto et al., 2021), canine cognitive dysfunction syndrome (Wu et al., 2023), and sporadic Creutzfeldt-Jakob disease (Schmitz et al., 2022).

Early studies revealed that peripheral blood NfL could serve as a biomarker for monitoring and tracking prognosis in AD (Rabl et al., 2024; Tsoy et al., 2024). Recent research underscores a strong correlation between plasma and CSF NfL levels, with significant changes observable during the preclinical phases of AD (Cai et al., 2023). Plasma NfL concentrations are crucial for detecting AD and its preclinical stages, such as subjective cognitive decline and mild cognitive impairment, as high concentrations have been associated with an increased risk of disease progression and cognitive decline (Ingannato et al., 2024). In patients with mild cognitive impairment, a rapid increase in plasma NfL is highly correlated with hippocampal atrophy, reduced brain glucose metabolism, and overall cognitive decline (Mattsson et al., 2019). Plasma NfL levels can also distinguish mutation carriers from non-carriers as early as 10–20 years before the expected onset of clinical symptoms (Hofmann et al., 2024). In autosomal-dominant AD, peripheral blood NfL changes occur more than a decade before the first clinical symptoms arise and have the potential to predict disease progression (Dominantly Inherited Alzheimer Network et al., 2019). Although the specificity of NfL in diagnosing AD is weak, elevated NfL levels can serve as a warning signal for AD, aiding in the screening of patients who require further diagnostic evaluation and the rational allocation of medical resources (Simonsen et al., 2023). In summary, while NfL may not be a specific blood biomarker for AD, it is essential for early diagnosis. It could be included as part of the memory clinic workflow, where it would need to be interpreted in the context of other clinical information.

#### Total tau

T-tau can serve as a biomarker for neurodegenerative injury, quantifying the degree of neuronal and axonal damage, and is closely related to neuronal damage during disease progression (Zhang et al., 2024b). CSF t-tau has been included in the international diagnostic framework as a key biomarker for diagnosing AD (Knudtzon et al., 2024). Although plasma t-tau lacks specificity for AD, its levels rise in the early stages of the disease and strongly correlate with CSF t-tau (Chiu et al., 2022).

Research indicates that plasma t-tau levels increase as AD advances, particularly during the transition from normal cognition to MCI and AD dementia (Park et al., 2022). A meta-analysis conducted by Ding et al. (2021) revealed that plasma t-tau levels were significantly elevated in individuals with AD compared to cognitively normal (CN) individuals, highlighting its potential as a tool for early AD detection before symptoms appear. However, a study using SIMOA technology found that while plasma t-tau achieved an AUC of 0.75 for differentiating AD from normal cognition, its performance was less effective (AUC < 0.60) in distinguishing MCI from normal cognition (Dong et al., 2023). Although plasma t-tau is a valuable marker of neurodegenerative changes, current measurements may capture both brain and peripheral tau proteins, limiting its accuracy in early AD diagnosis.

### Neuroinflammation related blood-based biomarkers

#### Glial fibrillary acidic protein

Astrocytes play a neuroprotective role in the early stages of neuroinflammation by providing nutrients, regulating the extracellular environment, and clearing harmful substances to repair damage. In the late stages of AD, astrocytes can form reactive astrocyte rings around Aβplaques, releasing inflammatory factors that exacerbate neuroinflammation and further damage neurons and synapses (Uddin and Lim, 2022). GFAP is an intermediate filament protein primarily expressed in astrocytes, with its encoding gene located on human chromosome 17 (Gao et al., 2022). When astrocytes are activated or damaged, the expression of the GFAP gene increases, leading to elevated GFAP production. Elevated plasma GFAP levels reflect astrocyte activation and neuroinflammation in the brain, and disruption of the neuroinflammatory microenvironment leads to subsequent synaptic dysfunction and cognitive impairment (Dark et al., 2024).

Plasma GFAP levels exhibit a strong correlation with cortical thinning (Montoliu-Gaya et al. 2023) and Aβ pathology (Koivumäki et al., 2024), while showing limited association with tau pathology (De Bastiani et al., 2023). Previous studies have demonstrated that GFAP in blood could serve as a candidate biomarker for early AD diagnosis (Hansen et al., 2022; Kim et al., 2023) and for predicting disease progression (Guo et al., 2023). In patients progressing from MCI to AD, plasma GFAP levels at baseline significantly increase (Silva‐Spínola et al., 2023). Research using SIMOA technology revealed increased plasma GFAP levels in individuals with prodromal AD and AD dementia compared with asymptomatic controls. These changes were associated with CSF Aβ levels and emerged roughly a decade before Aβ-PET positivity (Montoliu-Gaya et al., 2023). Plasma GFAP levels are strongly correlated with the onset of clinical AD, detectable more than a decade before diagnosis (O’Connor et al., 2023), making it an earlier biomarker than phosphorylated tau at threonine 181 (p-tau181). Significant changes in plasma GFAP levels can be observed ten years before the onset of clinical symptoms in patients with autosomal dominant Alzheimer’s disease (ADAD) (Johansson et al., 2023). Individuals carrying *ADAD* gene mutations exhibit significantly higher plasma GFAP concentrations than non-carriers (Johansson et al., 2023). However, plasma GFAP levels increase with age, so age should be considered when using GFAP for AD diagnosis (Ingannato et al., 2024).

Plasma GFAP levels rise ten years before clinical symptoms appear (Chatterjee et al., 2023), occurring after Aβ accumulation but before neurodegeneration and cognitive decline. Thus, GFAP holds excellent potential for detecting early Aβ deposition in AD, offering a promising option for early AD diagnosis.

#### Chitinase-3-like protein 1

Chitinase-3-like protein 1 (YKL-40) is a secreted glycoprotein that belongs to the glycoside hydrolase family 18 (Baldacci et al., 2017). It participates in various physiological and pathological processes, including extracellular matrix remodeling, inflammation, and tumor development (Fagan et al., 2021). YKL-40 is primarily associated with astrocytes in the brain and often indicates reactive neurotoxic diseases caused by neuroinflammation and stress-related pathways ((Yasuno et al., 2022; Connolly et al., 2023). YKL-40 is typically measured using ELISA and is one of the most extensively studied biomarkers associated with neuroinflammation (Cano et al., 2021; Schulz et al., 2021). The level of YKL-40 in the CSF of patients with AD and MCI is already elevated before clinical symptoms appear (Doroszkiewicz et al., 2023), confirming its involvement in the progression of early AD, particularly in relation to neuroinflammation and cerebrovascular dysfunction (Daniilidou et al., 2024). Moreover, YKL-40 levels in plasma are significantly elevated in AD patients, and higher plasma YKL-40 concentrations are associated with the pathological cascade of AD, which may promote Aβdeposition (Lananna et al., 2020). While YKL-40 in the blood has the potential to aid in the early diagnosis of AD, further research is needed to validate its clinical applicability and to elucidate its relationship with disease progression.

## Blood-based Biomarkers for Alzheimer’s Disease to Monitor Disease Progression

**Figure 4:**
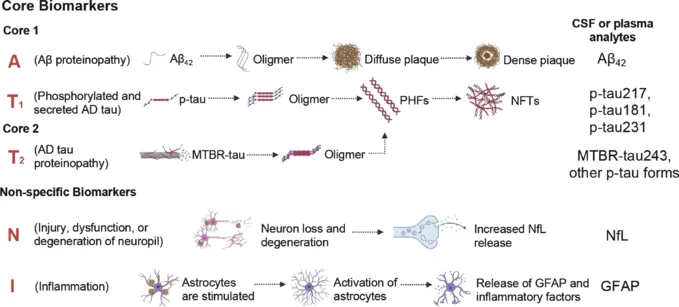
Pathological cascade and biomarker release mechanism diagram of AD. This figure summarizes the significant biomarkers involved in the pathophysiology of AD and their classification. Core biomarkers include: (1) Aβ pathology (A), characterized by decreased levels of Aβ_42_ in CSF or plasma, along with abnormal Aβ aggregation in the brain, leading to the formation of dense amyloid plaques; (2) Tau pathology (T), involving the abnormal aggregation of phosphorylated tau proteins (e.g., p-tau181, p-tau217, and p-tau231) and pathological isoforms of microtubule-binding region tau (MTBR-tau), such as MTBR-tau243. Non-specific biomarkers include: (1) Neuronal injury and degeneration (N), indicated by increased levels of NfL that reflect neuronal loss or axonal damage; (2) Neuroinflammation (I), characterized by the release of GFAP and other inflammatory factors following astrocyte activation. These biomarkers collectively reveal the complex pathological mechanisms of AD. Created in BioRender. Cui, Z. (2025) https://BioRender.com/ig82qw6. AD: Alzheimer’s disease; Aβ: amyloid-β; CSF: cerebrospinal fluid; GFAP: glial fibrillary acidic protein; MRI: structural magnetic resonance imaging; MTBR-tau: microtubule-binding region tau; NfL: neurofilament light chain; p-tau: phosphorylated tau; tau-PET: tau-protein positron emission tomography.

Over the past three decades, AD has evolved from symptom-based models to a continuum that includes clinical and biological features (Hampel et al., 2021b). Identifying biomarkers capable of dynamically tracking disease progression proves crucial for assessing transition speed from MCI to AD. Such assessments form the foundation for personalized treatment plans, notably since early intervention typically demonstrates more effective outcomes (Van Dyck et al., 2023). Biomarkers are one of the indispensable tools for evaluating the efficacy of AD treatment drugs (Hansson et al., 2023). The changes in biomarker levels during treatment intervention can indicate patients’ response to intervention therapy, thereby improving treatment strategies. The concentration and fold changes of biomarkers during follow-up are key indicators for monitoring disease progression and tracking cognitive trajectories of individuals with cognitive impairment (Jarek et al., 2024). **Figure 4** illustrates the relationship between the pathological cascade of AD and the release of biomarkers.

### Neurofilament light chain

The plasma NfL level significantly changes about 9 years before an AD diagnosis (Jia et al., 2024). NfL can reflect neuronal damage and axonal degeneration (Hansson et al., 2023). Research has shown that significant changes in plasma NfL are observed in the preclinical stage of AD, and a strong correlation exists between NfL concentration in CSF and plasma NfL levels (Cai et al., 2023). In AD patients, the average annual increase in CSF NfL levels is about 4%, which is associated with sustained neuronal damage during the progression of the disease (Knudtzon et al., 2024). Changes in plasma NfL levels are closely related to the degree of brain atrophy in AD patients; however, the relationship with Aβ deposition is insignificant, indicating its potential for evaluating cognitive decline (Hofmann et al., 2024).

A cohort study tracked plasma biomarker changes in AD patients from midlife (1993 to 1995) to late life (2011 to 2013) (Lu et al., 2024). It reported a decrease in Aβ_42/40_ by 0.005, an increase in p-tau181 by approximately 1.1 pg/mL, and an increase in NfL by 9.2 pg/mL. Among these, NfL demonstrated the strongest association with dementia onset (hazard ratio per 1-SD increase, 1.92; 95% CI: 1.72–2.14) (Lu et al., 2024).

Baseline plasma NfL concentrations increase across the AD spectrum, with patients exhibiting approximately a 5.9% annual elevation (Lehmann et al., 2023). Research involving CU, MCI, and AD dementia patients revealed progressively increasing NfL levels with disease advancement, showing yearly increases of 2.4 ng/L, 2.7 ng/L, and 4.9 ng/L, respectively (Mattsson et al., 2019). Plasma NfL is effective at predicting the current severity of the disease (Tsoy et al., 2024) and is comparable to standard imaging methods for assessing cognitive decline in Aβ-positive MCI patients (Mendes et al., 2024). This makes plasma NfL a simple, non-invasive way to monitor brain cell damage in AD.

Research has shown a slight increase in plasma NfL during early Lecanemab treatment, likely due to mild inflammation or cell injury resulting from Aβ clearance (Fan et al., 2023). With drug intervention, NfL levels decrease, indicating reduced neuronal damage. Thus, tracking changes in plasma NfL can help monitor Lecanemab treatment (Wojdała et al., 2023). This approach reveals early treatment effects and long-term outcomes, such as reduced neuronal damage. Moreover, monitoring how quickly NfL levels rise provides better predictive value than a single reading for determining whether MCI will progress to AD dementia (Wojdała et al., 2023). This highlights plasma NfL as a valuable marker for detecting subtle changes in neurodegenerative diseases.

### Phosphorylated tau217

A recent study suggests that plasma phosphorylated tau (p-tau217) can serve as a biomarker for Aβ pathology, with its elevation reflecting early Aβ deposition and tau protein entanglement in ADpatients (Jack et al., 2024). The level of plasma p-tau217 gradually increases with Aβ deposition, and these changes occur before the progression of cognitive impairment (Telser et al., 2022). Research demonstrates that among MCI patients progressing to AD dementia, p-tau217 levels increase annually by 0.79 ng/L, significantly exceeding the rates observed in non-converters (Mattsson-Carlgren et al., 2020). During the stages of AD dementia, p-tau217 concentrations peak and correlate closely with the severity of cognitive decline.

Pre-treatment and post-treatment suppression of tau pathological processes is accompanied by a significant reduction in p-tau217 levels in both blood and CSF. A decrease in p-tau217 in blood serves as a quantifiable indicator of therapeutic efficacy and can also act as a significant marker of disease progression (Wildsmith et al., 2024). Clinical trial findings indicate that lecanemab indirectly reduces tau pathology through plaque clearance (Wildsmith et al., 2024).

Regular measurement of p-tau217 levels enables tracking of pathological progression and the development of personalized therapeutic strategies. In summary, the dynamic changes in p-tau217 indicate the progression of AD from the preclinical to the clinical stages and constitute an essential biomarker for disease monitoring. Additionally, measuring p-tau in blood serves as an efficacy marker in lecanemab trials and helps reduce trial costs.

### Glial fibrillary acidic protein

The expression of GFAP is significantly altered in different neurodegenerative diseases; astrogliosis is a response of astrocytes to injury, reflecting their state (Bonomi et al., 2023). Studies have shown that GFAP concentrations increase from the preclinical to the dementia stages in AD, with levels in AD patients being up to 4% higher annually than in healthy controls in one biracial cohort study (Feng et al., 2023; Shen et al., 2023; Yakoub et al., 2023).

GFAP performs exceptionally well for forecasting dementia risk, with alterations potentially preceding tau pathology (Cicognola et al., 2021). Patients with MCI and AD display elevated GFAP concentrations in both CSF and blood compared to cognitively normal individuals (Nihashi et al., 2024). Elevated GFAP levels are associated with accelerated neurodegeneration rates (Feng et al., 2023) and predict future cognitive impairments in memory and language domains (De Meyer et al., 2024). Therefore, plasma GFAP functions as a predictor for future brain atrophy and cognitive decline in AD patients (Dark et al., 2024).

It is feasible to use plasma GFAP as a secondary endpoint in clinical trials (Abbas et al., 2025). Changes in plasma GFAP levels are significantly correlated with cognitive decline, and this progress is more pronounced in Aβ-positive populations. Notably, adequate Aβ plaque clearance through anti-Aβ immunotherapy substantially reduces plasma GFAP levels, establishing it as a compelling candidate biomarker for monitoring AD progression (Pontocorvo et al., 2022) and providing a quantifiable indicator of astrocyte response to anti-Aβ therapy.

## Blood-based Biomarkers for Differentiating Alzheimer’s Disease from Other Dementias

In the clinical diagnosis of neurodegenerative diseases, AD shares many clinical features with other forms of dementia, such as FTD, including memory loss and cognitive decline. Differentiating AD from other dementias is crucial for ensuring accurate diagnosis, personalized treatment, and care. This differentiation not only aids in optimizing patient selection for clinical trials and reducing the wastage of medical resources, but also provides patients with opportunities to participate in targeted therapeutic trials, thereby improving their quality of life and disease prognosis (Sun et al., 2025). In recent years, BBMs have shown significant potential as diagnostic tools in distinguishing AD from other dementias. **Additional Table 1** summarizes the main clinical manifestations and pathological proteins of neurodegenerative diseases (Bang et al., 2015; Borghammer et al., 2024; Fu and Halliday, 2025; Krishnamurthy et al., 2025; Ng et al., 2025).

**Additional Table 1:** Main clinical manifestations and pathological proteins of neurodegenerative diseases

Neurodegenerative disease	Main clinical manifestation	Core pathological protein	Reference
Alzheimer's disease	Episodic memory impairment, language deficits, visuospatial impairment, and executive dysfunction	Aβ, Tau	Krishnamurthy et al., 2025
Dementia with Lewy bodies	Fluctuating cognition, visual hallucinations, and Parkinsonism	α-Synuclein	Fu and Halliday, 2025
Parkinson's disease	Rest tremor, rigidity, bradykinesia, gait instability, and stooping posture	α-Synuclein	Borghammer et al., 2024
Frontotemporal dementia	Progressive deficits in behavior, executive function, or language	Tau, TDP-43, FUS	Bang et al., 2015
Vascular dementia	Executive dysfunction and stepwise deterioration	-	Ng et al., 2025

Aβ: Amyloid-β; FUS: fused in sarcoma; TDP-43: TAR DNA-binding protein 43.

### Amyloid-beta 42/40

Aβ_42_ is a biomarker for AD, while Aβ_40_ is elevated in various forms of dementia (Xu et al., 2022). In contrast, in other forms of dementia, such as vascular dementia (VaD), the plasma Aβ_42/40_ typically shows trivial changes or remains close to normal levels, with differences from healthy controls usually less than 1.5 times (Chouliaras et al., 2022). The plasma Aβ_42/40_ is highly effective in distinguishing AD from FTD (AUC = 0.88) and progressive supranuclear palsy (AUC = 0.78). However, its diagnostic performance in differentiating AD from dementia with Lewy bodies is limited, with an AUC of only 0.42 (Chouliaras et al., 2022). Although the difference in plasma Aβ_42/40_ between AD patients and healthy controls is smaller than that of other BBMs, such as plasma p-tau, the primary pathological features of VaD and FTD—vascular damage and frontotemporal degeneration, rather than Aβ plaque accumulation—suggest that plasma Aβ_42/40_ holds potential value in the differential diagnosis of AD and other dementias (Bun et al., 2023). To improve diagnostic accuracy, plasma Aβ_42/40_ is commonly used alongside other BBMs, such as plasma p-tau, to enhance diagnostic capacity in differentiating AD from other dementias.

### Phosphorylated tau217

AD patients exhibit substantially elevated plasma p-tau217 levels (a 4–6-fold increase; 150–300 pg/mL) compared with healthy individuals (50–70 pg/mL). Other dementias typically demonstrate less pronounced increases, generally remaining below 100 pg/mL (< 1.5 fold increase) (Chouliaras et al., 2022). In non-AD dementias such as FTD, negative p-tau217 results effectively exclude Aβ pathology, with a 90%–99% negative predictive value (Therriault et al., 2024).

A study comparing AD to other neurodegenerative conditions reported that plasma p-tau217 correctly identified 89% of AD cases (AUC = 0.96), demonstrating superior performance compared with plasma p-tau181, NfL, MRI-based cortical thickness in AD-related regions, and hippocampal volume (Palmqvist et al., 2020). Plasma p-tau217 showed no significant differences from CSF p-tau217 (Palmqvist et al., 2020). Electrochemiluminescence assay analysis indicated that plasma p-tau217 more effectively differentiated AD from FTD than p-tau181 (AUCs 0.93 *vs.* 0.91) (Thijssen et al., 2021).

A multinational study using Lumipulse and SIMOA platforms confirmed the exceptional accuracy of plasma p-tau217 in differentiating AD from other dementias, with AUC values of 0.952 and 0.955, respectively (Pilotto et al., 2024). This collective evidence establishes plasma p-tau217 as a highly effective tool for the differential diagnosis of Alzheimer’s disease.

### Glial fibrillary acidic protein

As an inflammatory biomarker, GFAP assists in differentiating AD from other dementias through distinct glial response manifestations. The range of plasma GFAP in ordinary people is 20–30 pg/mL, while the concentration of GFAP in AD patients is 60–90 pg/mL, an increase of 200%–300% (Shen et al., 2023). Other dementias demonstrate more constrained elevations of GFAP, typically ranging from 30 to 60 pg/mL (Chouliaras et al., 2022). A SIMOA-based study shows that plasma GFAP achieves an AUC of 0.81 for distinguishing AD from frontotemporal dementia, outperforming plasma Aβ_42/40_ and NfL (Chouliaras et al., 2022). Elevated serum GFAP levels are observed in AD spectrum disorders (e.g., posterior cortical atrophy) compared with non-AD conditions such as behavioral variant frontotemporal dementia (bvFTD) (Oeckl et al., 2022). Analysis using SIMOA technology reveals that AD patients exhibit nearly double the serum GFAP concentrations of bvFTD patients, achieving a differentiation AUC of 0.81 (Oeckl et al., 2022). Serum GFAP demonstrated 89% sensitivity and 79% specificity for distinguishing AD from bvFTD (Oeckl et al., 2019). These findings establish serum GFAP as an effective biomarker for differentiating AD from bvFTD, independent of CSF indicators.

## Blood-based Biomarkers for the Dynamic Monitoring of Neural Regeneration in Alzheimer’s Disease

**Figure 5:**
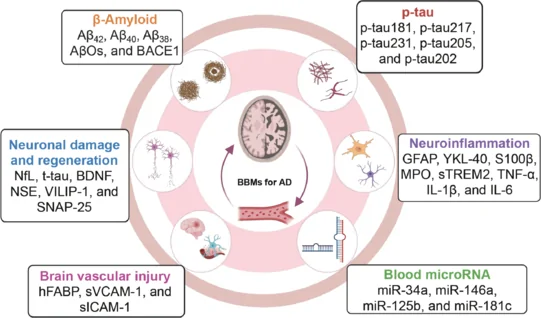
BBMs with potential application value for diagnosing AD. The biomarkers of AD can be categorized into several groups, including Aβ-related biomarkers, p-tau-related biomarkers, neuronal damage and regeneration-related biomarkers, neuroinflammation-related biomarkers, and brain vascular injury-related biomarkers. Additionally, blood microRNA has emerged as a novel biomarker for AD. Created in BioRender. Cui, Z. (2025) https://BioRender.com/gx0rwrd. AD: Alzheimer’s disease; Aβ: amyloid-β; BDNF: brain-derived neurotrophic factor; GFAP: glial fibrillary acidic protein; hFABP: heart-type fatty acid binding protein; IL-1β: interleukin-1β; IL-6: interleukin-6; MPO: myeloperoxidase; NfL: neurofilament light chain; NSE: neuron-specific enolase; p-tau: phosphorylated tau; S100β: central nervous system specific protein β; sICAM-1: soluble intercellular adhesion molecule-1; SNAP-25: synaptosomal-associated protein 25; sTREM2: soluble triggering receptor expressed on myeloid cells 2; sVCAM-1: soluble vascular cell adhesion molecule-1; TNF-α: tumor necrosis factor-α; VILIP-1: visinin-like protein 1.

Nerve regeneration is a process that repairs damaged nerves by regenerating nerve cells and restoring nerve tissue. As a prominent study of AD pathological features, recent evidence shows that nerve regeneration is an essential mechanism in diagnosing and treating AD (Gao and Li, 2023). There is some evidence that inducing neurogenesis and neuroplasticity may slow the progression of AD and restore basic abilities and some of the deficits in the brain (Zeng et al., 2024). These processes include the proliferation and differentiation of neural stem cells and the plasticity of synaptic connections. In AD, the regenerative capacity of neural stem cells is initially enhanced in the early stages of the disease but gradually declines as the disease progresses, leading to an irreversible decline in cognition (Boggio et al., 2012). These therapies need to be tested in clinical settings, and their success depends on the ability to monitor them. Neural repair-related BBMs could provide a quantifiable, dynamic measure of endogenous repair mechanisms, offering a novel, non-inflammatory way to monitor regeneration processes and assess the effectiveness of therapeutic agents in AD. **Figure 5** summarizes the biomarkers with potential application value for diagnosing AD.

### Brain-derived neurotrophic factor

Neurotrophins can regulate neurogenesis, synaptic plasticity, and neuronal survival. Key neurotrophins include BDNF, nerve growth factor (NGF), and glial cell line-derived neurotrophic factor (GDNF) (Jahan et al., 2026). Decreased circulating concentrations of neurotrophins are associated with the progression of AD, reflecting impaired nerve regeneration. A reduced level of BDNF can indicate Aβ and tau pathology, while a decrease in NGF signifies the deterioration of cholinergic function; GDNF supports the survival and function of dopaminergic neurons (Allen et al., 2013).

BDNF, a critical neurotrophic factor for synaptic plasticity and neuronal survival, has been shown to be differentially expressed between AD patients and healthy controls, with no significant differences found in NGF or GDNF (Eitan et al., 2023). A study indicated that AD patients have reduced circulating BDNF levels compared with healthy controls (Wang et al., 2024). A recent study supports the diagnostic potential of blood-based neurotrophic factors for AD (Zeng et al., 2024). In the study by Zeng et al. (2024), BDNF was included as one of the plasma biomarkers evaluated by the NULISAseq CNS disease panel. BDNF was significantly downregulated in AD, particularly in the blood of patients, where the reduction was the most significant among all neurotrophic factors, yielding an AUC of 0.76, sensitivity of 70%, and specificity of 66% (Asadi et al., 2024). This strong association links the reduction of BDNF to pathological amyloid deposition, making it a promising biomarker for the dynamic monitoring of neural regeneration in AD.

Blood BDNF levels can be influenced by numerous variables such as sex, age, and depressive symptoms (Zeng et al., 2025). For example, BDNF levels fluctuate between healthy individuals and those with dementia as they age. Furthermore, females and males also demonstrate differences in BDNF levels (Zeng et al., 2025). These factors present potential confounds that may decrease the reliability of blood BDNF as a biomarker for AD diagnosis and neural regeneration. In both contexts, the specific utility of BDNF as an AD diagnostic tool and as a monitor of neural regeneration should be carefully considered. Any clinical translation of this marker and its routine use in AD management would require validation in large, multicenter cohorts.

### Neurofilament light chain

NFL is a core subunit of neurofilaments, which combines with two other analogous proteins, neurofilament medium (NFM) and neurofilament heavy (NFH), to form the backbone of the neuronal cytoskeleton. This tripartite structure confers structural stability to the axon and functionally supports the integrity of neuronal signal transduction (Sharma et al., 2024). NFL is dysregulated and accumulates in a wide range of neurodegenerative conditions. NfL is closely related to progressive axonal degeneration (Sharma et al., 2024). Chronically elevated NFL levels are a hallmark of cumulative axonal injury that is both long-standing and non-resolving, indicating a perturbed regenerative niche, either due to a lack of neurotrophic signaling/reparative cues or due to inflammation-mediated suppression of reparative processes (Thal et al., 2024).

Elevated NFL is tightly associated with neural damage; however, it is possible that NFL dynamics could also be relevant to neural regeneration, particularly as regenerative processes involve some axonal remodeling (Khalil et al., 2024). However, NFL elevation is not disease-specific and is increased in multiple types of neural injury. NFL levels alone cannot be used to define the etiology behind the neural injury observed (McDowall et al., 2024). A decrease in NFL only indicates that the rate of ongoing axonal injury has declined, rather than that regeneration is occurring (Sharma et al., 2024). Definitive evidence of neural regeneration would require a combination with direct markers of reparative processes, such as BDNF. While NFL is a key biomarker for reflecting neurodegeneration, a multimodal biomarker approach is needed to interpret the results in the context of regeneration.

## Laboratory Assays of Blood-based Biomarkers for Alzheimer’s Disease

Plasma biomarkers are nearing clinical application for diagnosing and prognosing AD, mainly due to the availability of highly sensitive assays, particularly advancements in ultrasensitive immunoassays and mass spectrometry (Brand et al., 2022; Hunter et al., 2024). AD biomarkers and their detection methods are anticipated to be ready for clinical implementation in the coming years, facilitating clinical practice and interventional trials (Teunissen et al., 2022). Currently, various immunoassay techniques are used to detect AD biomarkers, including SIMOA, electrochemiluminescence immunoassay (ECLIA), and chemiluminescent enzyme immunoassay (CLEIA) (Janelidze et al., 2023; Peng et al., 2025). These methods, each with unique signal output mechanisms, strengths, and limitations, play crucial roles in AD diagnosis. Comparative studies have evaluated the diagnostic performance of these techniques in detecting biomarkers (Janelidze et al., 2021, 2023; Schindler et al., 2024). **Box 1** summarizes the detection principles of the AD biomarker detection technologies.

Box 1: Blood-based biomarker detection technology for Alzheimer’s disease.

(1) Single-molecule array (SIMOA) is a highly sensitive immunoassay method based on microarray chips. Compared to enzyme-linked immunosorbent assay, SIMOA has a sensitivity that is 100 to 1000 times greater and can accurately measure proteins in blood at extremely low concentrations (fg/mL). This method involves mixing magnetic beads coated with capture antibodies with the sample. The target protein is captured onto the magnetic beads and binds to a fluorescently labeled detection antibody. Subsequently, the magnetic beads are dispersed into the microholes on the microarray chip, with each microhole containing only one magnetic bead. Adding an enzyme substrate to generate a fluorescence signal will produce a strong fluorescence spot if a magnetic bead in the microhole is bound to the target molecule. The number of microholes with fluorescence signals can be counted to achieve digital absolute quantification at the single-molecule level.

(2) In electrochemiluminescence immunoassay, antibodies or antigens are labeled with the luminescent agent tris(2-pyridyl) ruthenium, while tripropylamine acts as the electron donor. Under the influence of voltage, an electrochemical reaction occurs, generating specific light signals. The magnetic particles serve as the solid-phase carrier, adsorbing the antibodies or antigens and reacting with the labeled molecules on the electrode surface, which produces continuous light signals.

(3) Chemiluminescent enzyme immunoassay uses enzyme-labeled antibodies or antigens (such as horseradish peroxidase or alkaline phosphatase) to catalyze a substrate reaction that generates chemiluminescent signals. The enzyme catalyzes the conversion of the substrate into a high-energy luminescent product, which releases photons upon returning to its ground state. The luminescent signal is directly proportional to the analyte concentration. This signal is captured by a photodetector and converted into an electrical signal, which is then used to quantify the antigen or antibody via a standard curve.

(4) Immunoprecipitation-mass spectrometry combines the advantages of immunoprecipitation and mass spectrometry, providing a high-throughput method for the quantitative and qualitative analysis of proteins in blood. This technology first separates substances, such as plasma, and sends them to a mass spectrometer after elution. Subsequently, the target protein is digested by enzymes into smaller peptide segments. Quantitative analysis measures the mass-to-charge ratio of the characteristic peptide segment of the target protein.

(5) Liquid chromatography-tandem mass spectrometry is a powerful molecular analysis tool that combines the high separation capability of liquid chromatography with the sensitivity and selectivity of mass spectrometry. The samples are first separated by liquid chromatography and then ionized and analyzed by mass spectrometry. In tandem mass spectrometry, target ions are fragmented, and the resulting fragment ions are analyzed to determine the composition and structure of the molecules.

Receiver operating characteristic (ROC) analysis is vital for evaluating the performance of diagnostic tests, with the AUC summarizing overall diagnostic accuracy. ROC analysis demonstrates that CSF proteins effectively discriminate between different stages of AD progression. With advancing biomarker research, plasma proteins show significant potential for identifying AD-associated abnormalities in CSF or PET imaging. Plasma Aβ_42/40_ and p-tau levels specifically reflect key pathological features of AD (Pereira et al., 2021; Teunissen et al., 2022). Multiple studies employ various experimental methods to detect AD biomarkers, benchmarking their performance against Aβ-PET or CSF Aβ_42/40_ to identify Aβ-positive cases (Hu et al., 2022; Allué et al., 2023). These findings provide substantial evidence supporting the clinical application of biomarkers.

### Diagnostic performance of plasma amyloid beta 42/40 assays in differentiating amyloid-beta positron emission tomography status

**Figure 6:**
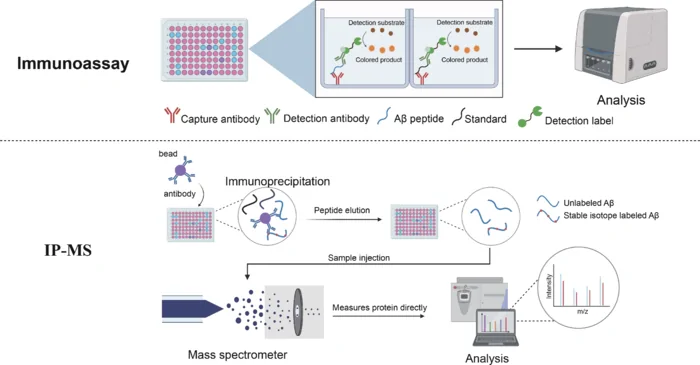
Comparison of the detection principles of two common plasma Aβ measurement methods. The two standard methods for measuring plasma Aβ are immunoassay (top) and IP-MS assay (bottom). In immunoassays, Aβ species are indirectly measured through antibody binding, and different detection antibodies must be used for each Aβ subtype. The immunoassay shown in this figure is based on a sandwich immunoassay using a flat panel. An immunoassay is quantified using external standards. In IP-MS analysis, the detector directly measures Aβ species and quantifies them using stable isotope-labeled Aβ internal standards. Created in BioRender. Cui, Z. (2025) https://BioRender.com/1kxwoi6. Aβ: Amyloid-beta; IP-MS: immunoprecipitation mass spectrometry.

The IP-MS assay developed by the University of Washington performed better than other assays. Four assays had an AUC of 0.845, while another had an AUC of less than 0.8 (Janelidze et al., 2021). In a comparative analysis of plasma amyloid beta 42/40 detection methods (Figdore et al., 2024), the IP-MS technique from the University of Washington again exhibited the highest diagnostic accuracy, with an AUC of 0.84. In contrast, the CLEIA and SIMOA methods achieved AUCs of 0.81 and 0.57, respectively (Figdore et al., 2024). In mass spectrometry-based assays, antibody-free liquid chromatography-tandem mass spectrometry (Ab-free LC-MS/MS) assays also performed well, with AUCs of 0.78 (Allué et al., 2023) and 0.87 (Pascual-Lucas et al., 2023). In a study comparing six plasma amyloid beta 42/40 experimental methods (three ligand-binding and three mass spectrometry-based methods) in a cohort of Alzheimer’s disease patients, the average AUC of mass spectrometry-based assays was 0.724, while the average AUC of ligand-binding assays was 0.672 (Zicha et al., 2023). In conclusion, the IP-MS assay demonstrates superior diagnostic accuracy, consistently achieving higher AUC values than other plasma amyloid beta 42/40 detection methods, making it the preferred choice for clinical applications in Alzheimer’s disease research and diagnostics. **Figure 6** shows the detection principles of two methods for measuring plasma amyloid beta.

### Diagnostic performance of plasma amyloid-beta 42/40 assays in differentiating cerebrospinal fluid amyloid-beta 42/40 status

In a head-to-head comparison based on CSF Aβstatus as a diagnostic criterion, the AUC of IP-MS for diagnosing AD was 0.855, while the AUCs of ECLIA, ELISA, and SIMOA were 0.778, 0.697, and 0.687, respectively (Janelidze et al., 2021). Furthermore, the IP-MS assay demonstrated exceptional performance in classifying CSF Aβ status across diverse cohorts, achieving an AUC of 0.92 in subjects with both unimpaired and impaired cognition (Li et al., 2022). Similarly, the fully automated Lumipulse G600II system, based on CLEIA, consistently exhibited robust diagnostic accuracy, with mean AUCs of 0.854 across multiple studies (Dakterzada et al., 2024; Morgado et al., 2024; Silva-Spínola et al., 2024) and exceptionally high AUCs in specific clinical cohorts, such as 0.907 for MCI and AD patients (Morgado et al., 2024) and 0.89 in CU individuals (Martínez-Dubarbie et al., 2023). In summary, based on IP-MS and CLEIA technology, these assays perform superiorly in detecting Aβ pathology, supporting their use as reliable tools for the clinical diagnosis and research of AD. Future research should further validate these methods in multiethnic and large-scale populations to improve their universality.

### Diagnostic performance of plasma p-tau assays in differentiating amyloid-beta positron emission tomographystatus

The detection of plasma p-tau181 based on SIMOA technology demonstrated varying diagnostic performance across different subject groups, with an AUC value of 0.805 for distinguishing between CU Aβ^–^ and CU Aβ^+^ individuals, 0.858 for differentiating between CU Aβ^–^ and mild cognitive impairment (MCI) Aβ^+^ individuals, and 0.920 for distinguishing between AD Aβ^+^ and CU Aβ^–^ individuals (Chatterjee et al., 2023). SIMOA-based plasma p-tau217 also exhibited high diagnostic accuracy for Aβ-PET status in two cohorts, with AUCs of 0.92 and 0.93 (Ashton et al., 2024). In a comparative study of two SIMOA assays, consistent AUCs of 0.92 were reported for identifying Aβ-PET positivity (Therriault et al., 2024). In a comparative analysis of six plasma p-tau assays, plasma %p-tau217 (the ratio of p-tau217 to non-phosphorylated tau) and p-tau217 measured by liquid chromatography-tandem mass spectrometry (LC-MS/MS) achieved AUCs of 0.927 and 0.916, respectively, outperforming other assays with AUCs below 0.9 (Schindler et al., 2024). An extensive cohort study involving 1422 blood samples further validated the diagnostic utility of plasma %p-tau217, showing an AUC of 0.97 for predicting Aβ-PET status in cognitively impaired individuals (MCI or AD), with 90% overall accuracy, 91% PPV, and 89% negative predictive value (NPV). The use of dual cut-off values improved these metrics to 95% overall accuracy, 95% PPV, and 96% NPV, surpassing the performance of a single cut-off and matching or exceeding that of an FDA-approved CSF assay (Barthélemy et al., 2024). The ability to detect plasma p-tau using SIMOA and LC-MS/MS technologies is essential for the clinical translation of blood markers and detection techniques. Moreover, it has demonstrated great potential as a substitute for Aβ-PET in diagnosing early AD.

### Diagnostic performance of plasma p-tau assays in differentiating cerebrospinal fluid amyloid-beta 42/40 status

The mass spectrometry-based plasma p-tau217 assay demonstrated superior performance in detecting abnormal CSF Aβ_42/40_ status (AUC = 0.947) and predicting progression to AD dementia (AUC = 0.932) compared to other plasma p-tau biomarkers (Janelidze et al., 2023). In a direct comparison of p-tau immunoassays, SIMOA-based plasma p-tau181 and p-tau217 assays yielded AUCs of 0.89 and 0.88, respectively, for identifying CSF Aβ_42/40_ positivity, while CLEIA-based assays showed AUCs of 0.84 for p-tau181 and 0.87 for p-tau217 (Ashton et al., 2023). Additionally, plasma p-tau/Aβ_42_ also identified CSF Aβ_42/40_ positivity well. Among the three studies using a fully automated Lumipulse G600II System (based on CLEIA) to detect plasma p-tau181/Aβ_42_ for identifying CSF Aβ_42/40_ positivity, the AUCs were 0.83 (Silva-Spínola et al., 2024), 0.870 (Dakterzada et al., 2024), and 0.79 (Martínez-Dubarbie et al., 2023), respectively. In a specialized memory clinic study, the fully automated Lumipulse G600II System (based on CLEIA) showed an AUC of 0.95 for plasma p-tau217/Aβ_42_ in identifying CSF Aβ_42/40_ positivity (Arranz et al., 2024). Advanced platforms such as the Lumipulse G600II system further enhance their clinical utility for early AD detection. Future research should focus on optimizing plasma p-tau detection technologies and validating their clinical utility, aiming to advance their application as an efficient tool for early AD diagnosis, thereby facilitating precise interventions and dynamic monitoring of DMTs.

### Diagnostic performance of combined blood-based biomarkers in differentiating amyloid-beta status

The progression of AD involves multiple biological processes, such as Aβ deposition, tau protein phosphorylation, neuroinflammation, and neuronal loss. Due to the complexity of its pathological mechanisms, a single blood biomarker is insufficient to comprehensively reflect disease information and diagnose AD. A study on AD patients showed that the combination of Aβ_42/40_ and p-tau217 achieved an AUC of 0.911 in distinguishing Aβ^+^ from Aβ^–^, which was superior to using plasma p-tau217 alone (AUC = 0.896) and Aβ_42/40_ alone (AUC = 0.787) (Schindler et al., 2024). In a study targeting CU participants, a multi-biomarker combination of plasma Aβ_42/40_, p-tau181, NfL, and GFAP produced an AUC of 0.91, outperforming any single biomarker in distinguishing Aβ^+^ from Aβ^–^ (Chatterjee et al., 2023). Integrating multiple BBMs can reduce false positive and false negative diagnosis rates, especially in the early stages of the disease. Therefore, combining various BBMs is significant for improving diagnostic accuracy and sensitivity.

Another study involving AD patients revealed that the combination of plasma Aβ_42/40_ and p-tau181 achieved an AUC of 0.871, and adding plasma NfL slightly increased the AUC to 0.871 with no significant change, while adding plasma GFAP raised the AUC to 0.873 (Schindler et al., 2024). A study involving participants with CU and MCI found that adding plasma NfL or GFAP to the diagnostic model did not significantly improve the accuracy of distinguishing Aβ^+^ from Aβ^–^ (Schindler et al., 2024), as plasma NfL and GFAP are more suitable for monitoring disease progression.

The increase in plasma NfL concentration in older adults will gradually accelerate, while the GFAP level in women rises faster than in men (Yakoub et al., 2023). Therefore, it is necessary to integrate AD risk factors such as age, gender, and APOE genotype into BBM-based diagnostic models. Incorporating these risk factors improved the ability of biomarker models to distinguish between Aβ^+^ and Aβ^–^ individuals (Chatterjee et al., 2023). The AUC increased to 0.92 in CU subjects and 0.98 in MCI patients (Chatterjee et al., 2023). A cross-sectional study involving ethnically diverse cohorts confirmed that including AD risk factors improved predictive accuracy for preclinical AD pathology. Among non-demented individuals, the curve increased from 0.938 to 0.948; in the MCI group, the improvement was more substantial, rising from 0.885 to 0.914 (Niimi et al., 2024). Integrating combined BBMs substantially enhances diagnostic accuracy for AD and enables earlier and more precise identification across varied clinical and demographic populations.

## From Blood-based Biomarkers to Targeted Therapies in Alzheimer’s Disease

### Therapeutic targets

Pharmacotherapy for AD comprises two main approaches: symptomatic therapies, which improve cognitive function, behavioral symptoms, or daily functioning without altering disease progression, and DMTs, which target core pathophysiological processes to delay or halt AD advancement (Iqbal et al., 2024). Currently, the only approved DMTs are anti-Aβ monoclonal antibodies. A transformative frontier in AD research lies in BBMs, which facilitate earlier and less invasive diagnosis and elucidate mechanistic insights into disease pathogenesis, thereby unveiling novel therapeutic targets with high translational potential (Zhang et al., 2025). In the pathological progression of AD, Aβ_42_ exhibits a pronounced propensity for aggregation into Aβ plaques, whereas Aβ_40_ participates minimally in plaque formation. The plasma Aβ_42/40_ ratio is a dynamic biomarker reflecting cerebral Aβ metabolic dysregulation. Specifically, elevated Aβ_42_ production or impaired clearance in the brain correlates with a reduced plasma Aβ_42/40_ ratio, a quantitative signature strongly associated with AD risk and disease progression. **Additional Table 2** summarizes some key clinical trials of anti-Aβ drugs for AD (Van Dyck et al., 2023; Sims et al., 2023; Salloway et al., 2025).

**Additional Table 2:** Key phase III clinical trials of anti-amyloid-β therapy for Alzheimer's disease (AD)

Study (NCT Number)	Design	Primary outcome measures	Results	Significance	Reference
A Study to Confirm Safety and Efficacy of Lecanemab in Participants with Early AD (NCT03887455).	Randomized, double-blind, placebo-controlled, parallel-group study.	Change in Clinical Dementia Rating-Sum of Boxes at 18 months.	Lecanemab 1.21 *vs*. placebo 1.66 (*P* <0.001).	One of the first trials to demonstrate significant slowing of cognitive decline in early AD with amyloid-targeted therapy.	Van Dyck et al., 2023
A Study of Donanemab (LY3002813) in Participants with Early AD (NCT04437511).	Randomized, double-blind, placebo-controlled trial.	Change in Integrated AD Rating Scale at 76 weeks.	(1) Low/medium tau population: Donanemab -6.02 *vs*. Placebo -9.27 (*P* < 0.001). (2) Combined population: Donanemab -10.19 *vs*. Placebo-13.11 (*P* <0.001).	Demonstrating robust amyloid clearance and cognitive benefit, supporting donanemab as a high-efficacy anti-amyloid agent.	Sims et al., 2023
A Study of Donanemab (LY3002813) Compared with Aducanumab in Participants with Early Symptomatic AD (NCT05108922).	Randomized, open-label, parallel-group study.	Change in Integrated AD Rating Scale at 76 weeks.	(1) Overall population: Donanemab 37.9% *vs*. Aducanumab 1.6% (*P* < 0.001). (2) Low/medium tau population: Donanemab 38.5% *vs*. Aducanumab 3.8% (*P* = 0.008).	The first direct comparison of two anti-amyloid antibodies shows faster and more complete amyloid clearance with donanemab.	Salloway et al., 2025

Clearing AβOs is currently the focus of research on treating AD, such as with lecanemab. In a pivotal study evaluating the therapeutic efficacy of lecanemab in early AD (McDade et al., 2022), patients receiving biweekly 10 mg/kg lecanemab demonstrated significant reductions in cerebral Aβ plaque burden at 12 and 18 months, accompanied by attenuated clinical cognitive decline. Notably, the lecanemab group exhibited a statistically significant increase in the plasma Aβ_42/40_ ratio compared with placebo. Strikingly, changes in plasma Aβ_42/40_ showed a robust inverse correlation with PET-measured Aβ load reduction, indicating synchronous peripheral biomarker shifts with central Aβ clearance. These findings mechanistically substantiate that the clinical benefits of lecanemab are mediated through targeted engagement of cerebral Aβ pathology (Van Dyck et al., 2023).

Inhibiting the production of the Aβ protein is another important target for treating AD. ALZ-801 (valiltramiprosate) (Hey et al., 2024), an orally administered and highly brain-penetrant disease-modifying agent, selectively disrupts the formation of neurotoxic AβO to mitigate Aβ-driven neurodegeneration. Phase 2 clinical data demonstrated that ALZ-801 treatment significantly reduced hippocampal atrophy rates and stabilized cognitive function. Intriguingly, plasma Aβ_42_ levels exhibited a biphasic response—an initial transient increase (+Δ13.6% at week 13) followed by a sustained decline (−Δ4.2% at week 104)—suggesting enhanced Aβ_42_ efflux from the brain into peripheral circulation, coupled with subsequent systemic clearance. The observed dissociation between declining plasma Aβ_42_ and stable Aβ_40_ levels further indicates a drug-induced shift in Aβ metabolic processing, preferentially reducing pathogenic Aβ_42_ accumulation. These biomarker dynamics confirm target engagement and provide mechanistic evidence for the disease-modifying potential of ALZ-801 to slow AD progression (Tolar et al., 2020). These findings collectively establish plasma Aβ_42/40_ as a robust peripheral biomarker reflecting Aβ dyshomeostasis. This ratio represents a convergent node connecting diverse therapeutic mechanisms, ranging from direct Aβ clearance to proteostatic modulation. The plasma Aβ_42/40_ ratio provides a key indicator for evaluating drug efficacy and Aβ metabolism targeting different pathways. However, changes in the plasma Aβ_42/40_ ratio are inconsistent with clinical effectiveness (Bittner et al., 2025). Not all therapies that reduce Aβ can significantly alter this ratio, reflecting the complex mechanisms of Aβ plaque clearance (Pontecorvo et al., 2022).

### Safety

AD is a chronic, progressive neurodegenerative disorder that typically necessitates extended, potentially lifelong pharmacotherapy. This prolonged duration of treatment raises concerns that even seemingly minor toxicities may, through cumulative exposure, result in significant adverse outcomes. Consequently, safety assessment data provide pivotal evidence for decision-making throughout the drug development lifecycle (Fan et al., 2022). Should investigations reveal substantial safety concerns during clinical trials, therapeutic candidates demonstrating considerable efficacy may nevertheless warrant discontinuation or fundamental redesign of the development program.

Currently, most drugs used for AD DMTs are still in clinical trials, and strict evaluation of safety is critical. It is essential to establish reasonable entry barriers to prevent the introduction of unsafe compounds into clinical environments (Ide et al., 2016; Morató et al., 2023). Furthermore, comprehensive safety profiling must extend across all development stages—from initial human trials through post-marketing surveillance. Regulatory agencies mandate ongoing pharmacovigilance even following market approval to detect delayed-onset adverse reactions or rare events potentially undetected during pre-approval clinical studies (Zhang et al., 2024a).

For therapeutic agents targeting Aβ clearance as the primary mechanism of action in AD, the adverse event profile extends beyond clinical manifestations such as headache, dizziness, hallucinations, and anxiety, necessitating particular vigilance for amyloid-related imaging abnormalities (ARIA) (Hampel et al., 2023). ARIA encompasses two distinct neuroimaging entities: ARIA-E (edema/effusion) and ARIA-H (hemosiderin deposition), both attributed to inflammatory-mediated compromise of vascular integrity, leading to extravasation of blood constituents and protein-rich fluid into the brain parenchyma (Yue et al., 2026). Radiologically, ARIA-E presents as parenchymal hyperintensities on T2/FLAIR MRI sequences, indicative of vasogenic edema or inflammatory exudates. Clinically significant recurrence of ARIA-E exacerbates vascular leakage, accelerating cognitive decline and neuroinflammation, with severe cases progressing to ataxia or impaired consciousness. Conversely, ARIA-H manifests as subcentimeter (< 10 mm diameter) punctate hypointensities on T2*/SWI sequences, reflecting microhemorrhages or hemosiderin deposits. These lesions may potentiate the burden of cerebral small vessel disease, contributing to superimposed vascular cognitive impairment on existing AD pathology and carrying a risk of life-threatening intracerebral hemorrhage (Gueorguieva et al., 2023). Meta-analytic evidence confirms that anti-Aβ monoclonal antibodies (e.g., lecanemab, donanemab) demonstrate significant efficacy advantages across endpoints, including ADCOMS, CDR-SB, ADAS-Cog14, and Aβ-PET burden, compared to placebo. Notwithstanding these therapeutic benefits, pharmacovigilance data reveal a 4.35-fold increased relative risk for overall ARIA incidence in treated cohorts, with statistically significant elevations observed for both ARIA-E and ARIA-H subtypes compared with controls (Wang et al., 2025).

Apolipoprotein E4 (ApoE4) carriers demonstrate more pronounced Aβ plaque clearance with anti-Aβ monoclonal antibody therapy but concurrently experience a 3–4-fold increased risk for ARIA (Millet et al., 2024). This paradoxical effect is due to the multifactorial mechanism of increased neuroinflammation and heightened blood-brain barrier permeability, which is linked to the APOE4 allele (Pires and Rego, 2023). The clinical approach should shift to a therapeutic strategy based on precise genetic stratification of patients. Furthermore, considering the patients’ physiology, the doses of these drugs should be adapted to their individual needs. For ApoE4-positive patients, the clinical course of the disease may be altered, which is not expected to necessitate a change in the dose (Zimmer et al., 2025).

Long-term use of anti-Aβ monoclonal antibodies can compromise the integrity of the blood-brain barrier, increasing the risk of cerebral edema and hemorrhage (Kim et al., 2025). Immune-mediated side effects include acute infusion-related reactions such as fever and chills, as well as localized inflammatory responses caused by the binding of the antibody to Aβ plaques in the brain. These side effects are critical to the clinical translation and application of Aβ-targeting drugs (Kolay et al., 2024). The adverse effects and potential iatrogenic risks associated with anti-Aβ monoclonal antibodies should not entirely overshadow the potential benefits of these treatments. Although recent advances in the development of AD therapies have been rapid, a clear definition of adverse effects and a comprehensive understanding of the safety profile of these treatments remain essential prerequisites for their implementation in clinical practice. The safest approach to introducing disease-modifying therapies for AD into clinical practice is to balance efficacy with safety.

### Clinical translation

The landscape of AD therapeutics has witnessed transformative advances in recent years, marked by the approval of anti-Aβ DMTs. Previously, five drugs—tacrine, donepezil, rivastigmine, galantamine, and memantine—were approved by the U.S. Food and Drug Administration (FDA) for the treatment of AD (Wu et al., 2023). The primary mode of action of these drugs is to improve clinical symptoms and delay disease progression. In June 2021, aducanumab received accelerated approval from the FDA as the first DMT; however, its clinical application has stalled due to controversies regarding efficacy and safety. Subsequently, lecanemab received FDA approval in January 2023, followed by donanemab in July 2024, further driving the clinical application of DMTs (Cummings et al., 2024). **Additional Table 3** summarizes the FDA-approved anti-Aβ drugs (Van Dyck et al., 2023; Salloway et al., 2025).

**Additional Table 3:** FDA-approved anti-amyloid-β (Aβ) drugs

Trade name	Drug generic name	Target	Administration	Clinical application	Major side effect	Reference
Leqembi	Lecanemab-irmb	Soluble Aβ fibrils	Intravenous infusion every two weeks	Early AD patients	ARIA-E/H, Cerebral edema and hemorrhage	Van Dyck et al., 2023
Kisunla	Donanemab-azbt	N3pG-Aβ aggregated plaques	Monthly intravenous infusion	Early AD patients	ARIA-E/H, Cerebral edema and hemorrhage	Salloway et al., 2025

AD: Alzheimer’s disease; FDA: Food and Drug Administration.

Lecanemab, an IgG1 monoclonal antibody targeting Aβ aggregates, was developed by Eisai in partnership with BioArctic and Biogen (Hoy, 2023). An accelerated Phase 3 Clarity AD trial validated its approval, demonstrating marked reductions in cerebral Aβ burden, which coincided with a statistically significant decrease in cognitive and functional decline in patients with early AD. Alongside the administration of treatment, adverse incidents (e.g., cases of ARIA) were documented, with the majority exhibiting asymptomatic or mild to moderate symptoms (Söderberg et al., 2023). The approval of lecanemab marks the first DMT to establish Aβ-targeted efficacy in a Phase 3 trial, confirming the therapeutic relevance of the Aβ cascade hypothesis. This therapy delays disease progression in early-stage patients, thereby prolonging the clinically relevant period of cognitive decline (Van Dyck et al., 2023). Further longitudinal studies are essential to fully elucidate the long-term benefit-risk profile of lecanemab in real-world populations.

Donanemab, an IgG1 monoclonal antibody developed by Eli Lilly and Company (Kang, 2024), selectively binds to Aβ deposits within mature plaques, facilitating substantial clearance of established amyloid pathology. The results of the TRAILBLAZER-ALZ-2 trial, a 76-week, multicenter, randomized, double-masked, placebo-controlled study, showed a statistically significant reduction in cerebral amyloid deposits, along with a statistically significant decrease in the rate of decline in both memory and function compared to placebo (Sims et al., 2023). Treatment effects were most evident in individuals with low-to-intermediate tau pathology at baseline. Donanemab therapy was associated with increased incidence rates of amyloid-related imaging abnormalities with edema (ARIA-E). A post hoc analysis indicated that the level of amyloid deposits at the start of treatment was directly related to the extent of reduction in amyloid deposits but inversely related to the likelihood of complete amyloid clearance (Shcherbinin et al., 2022). In cohorts achieving amyloid clearance and in brain regions affected later in AD progression, donanemab treatment significantly reduced pathological tau spread, providing a novel framework for reducing the long-term treatment burden while targeting core AD pathology.

Although lecanemab and donanemab represent transformative advances in disease-modifying therapies for AD, they do not signify the final achievements in clinical translation or therapeutic endpoints (Teipel et al., 2025). Subsequent large-scale longitudinal trials must fully characterize their long-term clinical benefits and safety profiles. These therapeutic breakthroughs have generated substantial demand for innovations in AD diagnostics, where current gold-standard positron emission tomography imaging and cerebrospinal fluid biomarker analyses prove insufficient for scalable population screening—an essential prerequisite for the implementation of precision therapies (Nakashima et al., 2025). BBMs are essential tools across the AD continuum, supporting early detection, etiology-specific diagnosis, real-time treatment monitoring, and prognostic evaluation. The continued development of BBMs will fundamentally advance the clinical translation of AD therapeutics.

## Limitations

While this review synthesizes pivotal advances in BBMs for AD, several inherent limitations constrain comprehensive coverage. This article mainly discusses Aβ, tau species (p-tau181/p-tau217), and neural regeneration biomarkers (such as BDNF and NfL), but excludes emerging biomarker categories. Emerging biomarkers, particularly synaptic vesicle proteins (such as synaptic binding protein-1), metabolic intermediates (such as bile acids), and inflammasome components, have shown high potential for application in independent cohort studies.

Additionally, although this review notes the importance of detection techniques such as SIMOA and IP-MS in AD diagnosis and compares them, there are still key gaps in the systematic cross-platform comparison of robustness analysis, especially under actual pre-analysis conditions involving sample stability, matrix effects, and isotype-specific antibody validation. Subsequently, meta-analyses of diagnostic techniques on different platforms are needed to better evaluate their application value. We underscore the potential of dynamically monitoring the processes of neural repair; however, longitudinal validation, which links temporal variations in BDNF and other BBMs to structural neural plasticity or intervention outcomes, demands prospective trials with coordinated efforts. Current research progress restricts the number of comprehensive reviews on this topic.

The studies and their cohorts included in this review exhibit significant geographic and racial distribution disparities, focusing predominantly on European and American populations. Differences in population distribution may limit the applicability of biomarker performance (such as cut-off values and diagnostic efficacy) and the main conclusions summarized in this article to other ethnic and regional populations. This article primarily reviews the progress of research on BBMs for AD in recent years. The controversies between various BBMs have not been fully discussed. Due to the rapid development of this field, there is no consensus on the best biomarkers and detection techniques for diagnosing AD. Various types of biomarkers are not absolutely superior or inferior, and comparing BBMs is a process of performance optimization and iteration. We believe that, in the near future, new biomarkers or technologies will emerge to address this debate and pave the way for BBMs to enter clinical applications. Finally, quantitative frameworks for incorporating multi-omic biomarker signatures into clinical staging systems are still underdeveloped. These limitations highlight emerging methodological and conceptual challenges rather than weakening the field’s progress, emphasizing the need for standardized biorepositories, consistent assay procedures, and multi-center validation initiatives.

## Conclusion and Perspectives

This review addresses the latest research progress on BBMs for AD and their multifaceted roles in improving diagnostic and treatment strategies. It systematically examines the transformative potential of BBMs in several key areas: early diagnosis, disease progression monitoring, differential diagnosis, dynamic assessment of the neural regeneration process, and innovation in biomarker detection technologies. This focus distinguishes this review from previous reviews on AD BBMs. We emphasize that core biomarkers in the blood, such as Aβ and p-tau, have been thoroughly validated as capable of identifying the early stages of AD. This review also covers the latest progress in DMTs, particularly anti-Aβ monoclonal antibodies, and discusses their translational potential in clinical applications. Integrating BBMs into clinical practice is vital for diagnosing and treating AD. In addition to summarizing the existing evidence, this review calls for interdisciplinary collaboration to accelerate the transformation of BBMs from research mechanisms to practical applications, thereby achieving personalized diagnosis and treatment of AD.

The emergence of BBMs has significantly advanced AD research and clinical practice, providing enormous application value for early diagnosis, disease monitoring, and differential diagnosis (Klyucherev et al., 2022). Biomarkers, including Aβ and p-tau proteins, can identify AD before clinical symptoms appear, offering essential opportunities for early intervention and proving crucial for the efficacy of potential treatment strategies. Moreover, accurate plasma p-tau217 measurement is sufficient for diagnosing AD, providing reliability comparable to approved CSF measurements in detecting abnormal Aβ-PET results (Jack et al., 2024). Integrating BBMs into clinical workflows can fundamentally change AD management by enabling more personalized and effective care. However, despite their advantages of simplicity, cost-effectiveness, and ease of repeated sampling, challenges remain in clinical applications. Different clinical scenarios require varying sensitivity and specificity, including early screening, disease diagnosis, progression prediction, and treatment monitoring (Bolton et al., 2025). Therefore, it is necessary to develop clear usage guidelines to assist clinicians in making informed decisions based on biomarker results. Future research should prioritize improving detection techniques and validating their clinical applicability through extensive studies, further exploring the transformative potential of BBMs in AD diagnosis and management (Bougea et al., 2025).

Research and clinical applications of BBMs in treating AD have rapidly progressed (Hunter et al., 2024). However, the lack of standardized analysis guidelines and technical protocols seriously hinders their comparability and applicability in different research and clinical environments. Differences in technical methods and reagent selection among research teams pose challenges for comparing and interpreting cross-study data. For example, detecting p-tau and the Aβ_42/40_ ratio relies on highly sensitive analytical methods such as ultra-high-performance liquid chromatography-mass spectrometry or immunoassay (Cano et al., 2021; Lu et al., 2024). However, variations in laboratory equipment performance, operating procedures, and quality control measures often lead to inconsistent results. Additionally, standardization of sample processing, including anticoagulant type, storage conditions, and centrifugation parameters, significantly impacts detection (Monteiro et al., 2023). These technological and procedural differences limit the diagnostic value of blood biomarkers and restrict the large-scale analysis of clinical data. Therefore, professional associations or international organizations must develop unified analytical guidelines covering all aspects of sample collection, storage, testing, and reporting. Moreover, it is also necessary to create internationally standardized reference materials, calibration procedures, and quality control agreements. Global multi-center collaborative research is crucial for verifying and optimizing inter-laboratory consistency and data reliability. Internationally recognized consensus guidelines, including BBM sampling methods, detection techniques, data reporting formats, and reference intervals, should be established to accelerate the clinical application of BBMs (Pan et al., 2023).

Although BBMs have made significant progress in diagnosing AD, most studies have been limited by small sample sizes, narrow geographic regions, and homogeneous populations (Silva-Spínola et al., 2024; Arranz et al., 2024). These limitations prevent the research results from being applicable across different races, age groups, and disease stages (Pan et al., 2023). Additionally, the current evaluation of the diagnostic performance of BBMs mainly relies on retrospective and single-center studies (Colvee-Martin et al., 2024; Schindler et al., 2024), which cannot fully reflect clinical applications in the real world. Multi-center, prospective studies are crucial for verifying the robustness and reproducibility of these biomarkers in different medical environments. Future research should prioritize establishing large-scale, multi-ethnic cohorts, determining the optimal combination of biomarkers, and verifying their effectiveness in these cohorts. By utilizing the complementary advantages of multiple biomarkers, diagnostic accuracy and the ability to predict disease progression can be significantly improved. Furthermore, long-term follow-up studies using BBMs combined with other diagnostic approaches, such as cerebrospinal fluid markers and neuroimaging techniques, are necessary to fully measure their clinical utility and translational potential.

## Additional files:

***Additional Table 1:*** *Main clinical manifestations and pathological proteins of neurodegenerative diseases.*

***Additional Table 2:*** *Key phase III clinical trials of anti-amyloid-*β *therapy for Alzheimer’s disease (AD).*

***Additional Table 3:*** *FDA-approved anti-amyloid-*β *(A*β*) drugs.*

## Data Availability

*All relevant data are within the paper and its Additional files.*
